# Supplementary material for: Influence of grain size on the solid-state direct reduction of polycrystalline iron oxide
Source: Commun Mater. 2026 Feb 18;7(1):82. doi: 10.1038/s43246-026-01106-z (PMC12995716; doi:10.1038/s43246-026-01106-z)
Supplement: Supplementary file 1 — Supplementary Information [file 43246_2026_1106_MOESM1_ESM.pdf]

Supplementary information

Influence of grain size on the solid-state direct reduction of polycrystalline iron oxide

Barak Ratzker[1,*], Martina Ruffino[1], Shiv Shankar[1], Yan Ma[1,2], Dierk Raabe[1]

[1] *Max Planck Institute for Sustainable Materials GmbH, Max-Planck-Str. 1, 40237 Düsseldorf, Germany*

[2] *Department of Materials Science and Engineering, Delft University of Technology, Mekelweg 2, 2628 CD Delft, the Netherlands*

* Corresponding author: b.ratzker@mpi-susmat.de

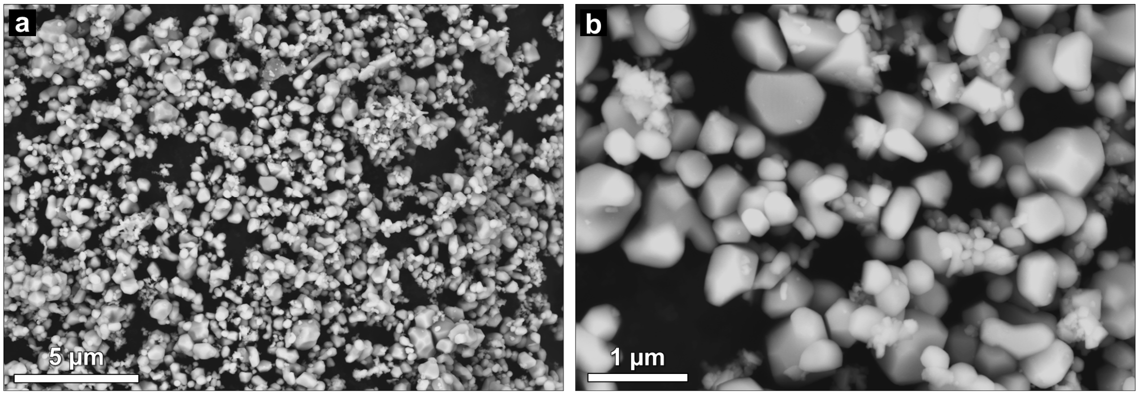

Supplementary Figure 1 | Morphology of hematite powder. BSE-SEM images showing the morphology of the hematite powder used for produced the sintered samples; (a) low and (b) high magnification. The particles are mostly spherical with a particle size ≤1 µm.

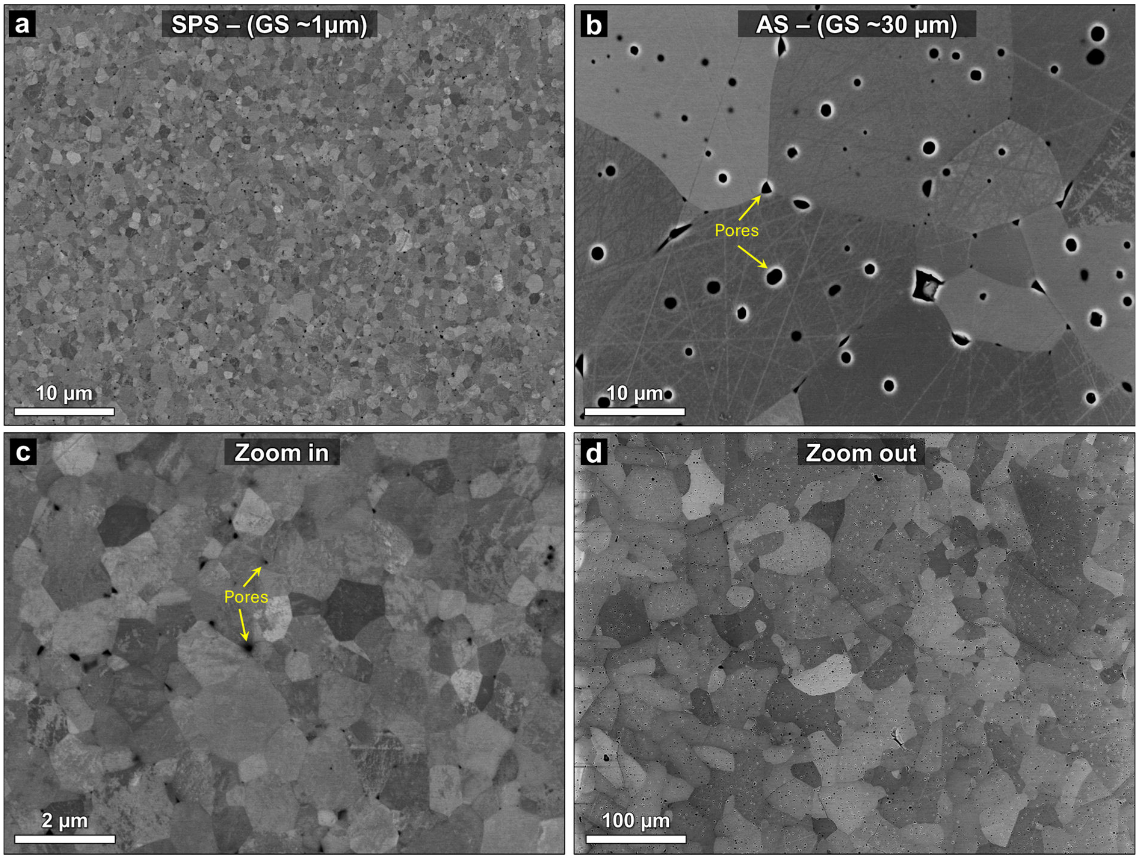

Supplementary Figure 2 | Microstructure of sintered hematite samples. BSE-SEM images showcasing the microstructures of the (a) ultrafine and (b) large grain size hematite samples produced by spark plasma sintering (SPS) and air sintering (AS), respectively. Further (c) high magnification of the ultrafine grains and (d) low magnification of the large grains. Sintering pores can be observed in both materials; in the large-grained sample they are either intragranular and spherical (~1–2 μm in size) or at triple points, while in the ultrafine-grained sample they are all nanometric and only found at triple points.

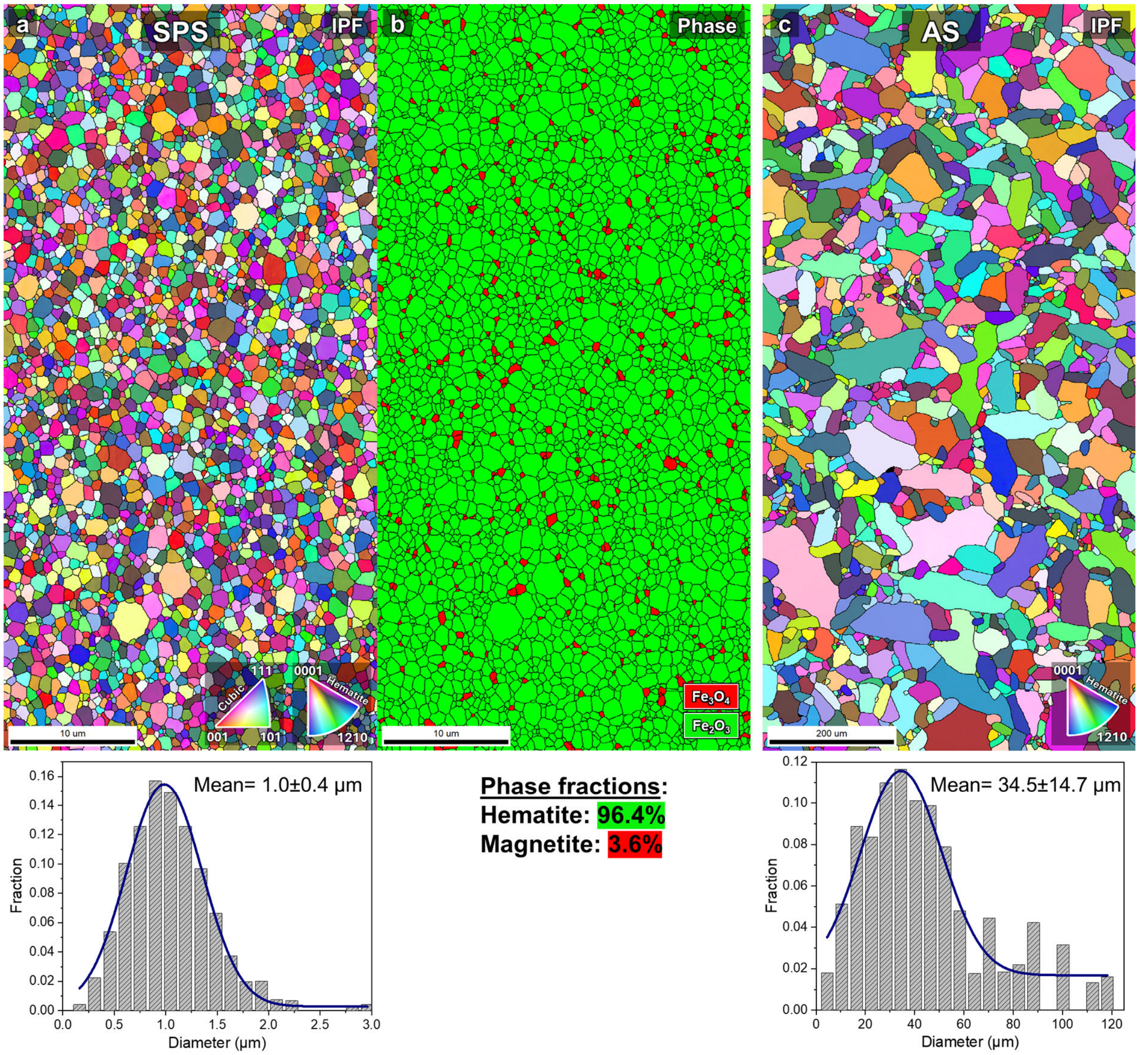

Supplementary Figure 3 | EBSD and grain size analysis of sintered hematite samples. EBSD analysis of (a) SPS ultrafine and (b) AS large grain size hematite, note that the scale bar in (b) is 20 times that in (a). In the SPSed ultrafine sample, as shown in the corresponding phase map, a magnetite content (~3.5%) is retained from the original iron oxide. The corresponding grain size distributions for each scan is presented below the IPFs.

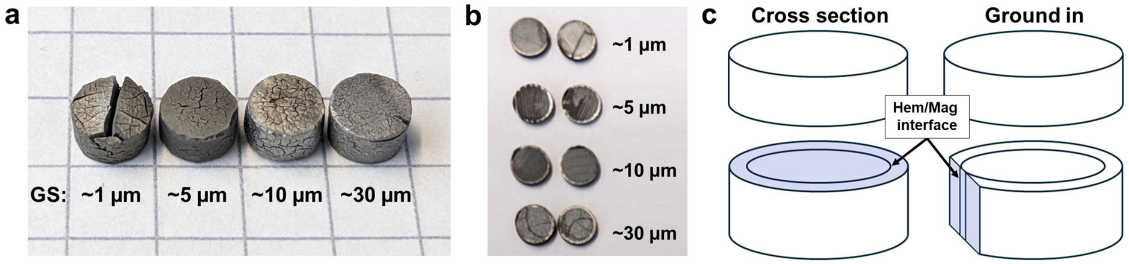

Supplementary Figure 4 | Reduced samples examination. Photographs of samples (a) after full reduction, 30 min holding at 700 °C and (b) after partial reduction for 1 min at 700 °C samples were cut in half across the circular cross-section. (c) The partially reduced microstructures were analyzed after metallographic preparation of the cross section or ground in surfaces, as shown schematically.

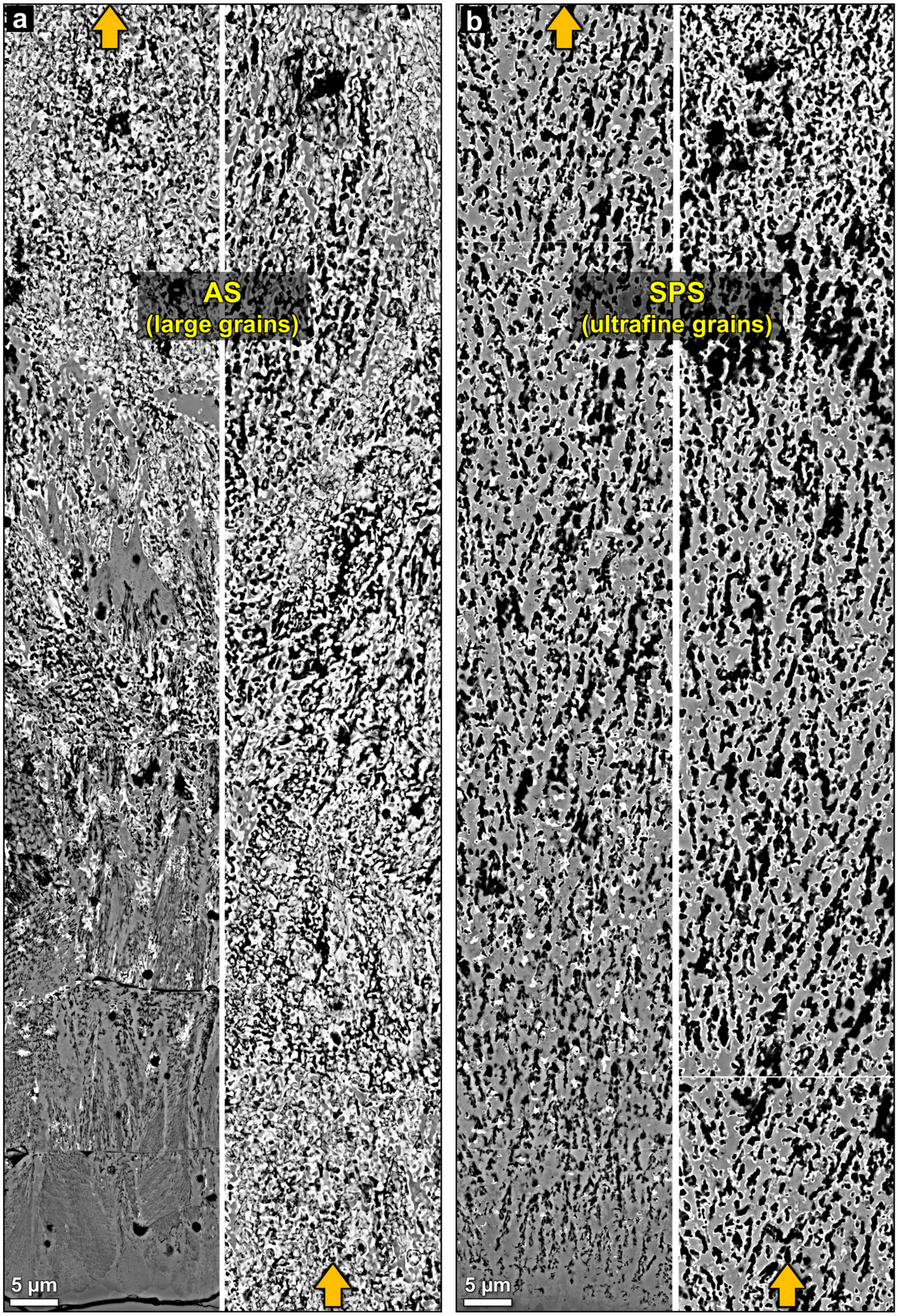

Supplementary Figure 5 | Microstructure of the reduced layers. BSE-SEM collage images showcasing most of the reduced layer in the (a) large- and (b) ultrafine-grained samples. The orange arrows depict the continuation of the compound images.

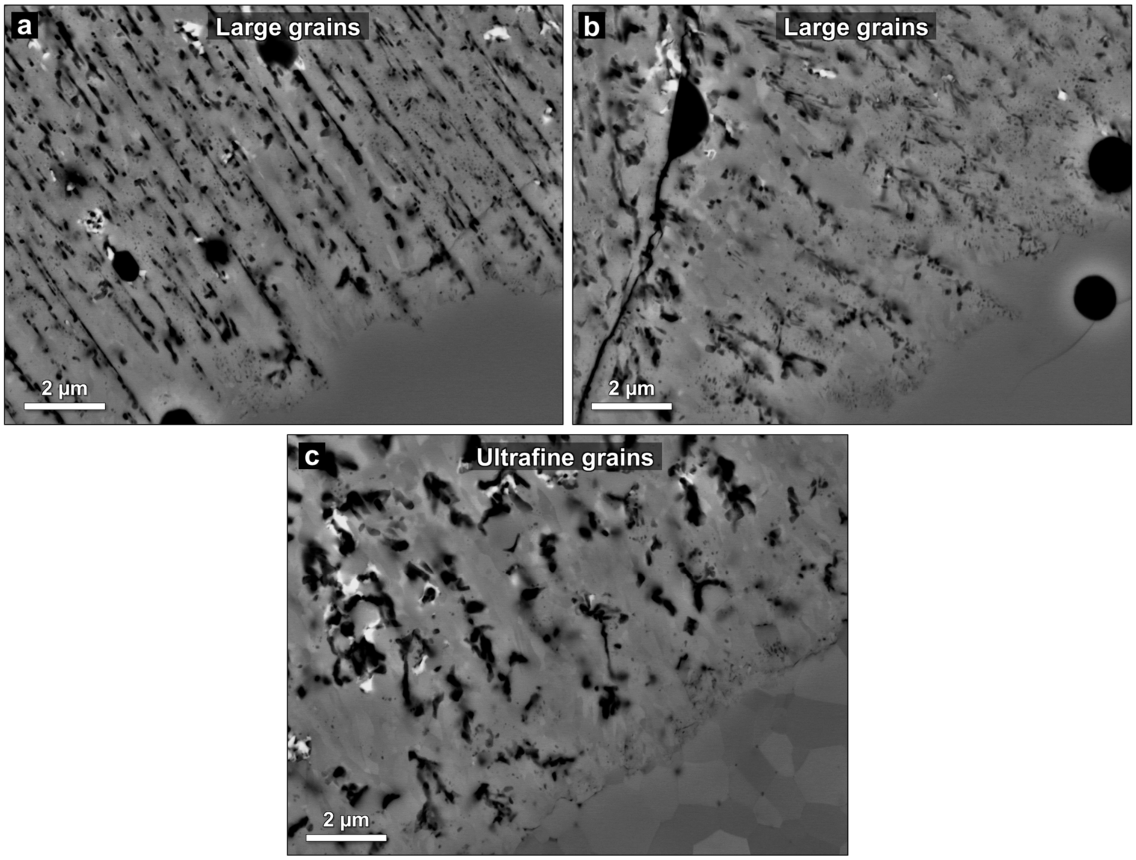

Supplementary Figure 6 | Porosity at the hematite/magnetite interface. BSE-SEM high magnification images of the hematite/magnetite interface in (a) large grains with strong directionality of pore channels, (b) large grains with poor directionality, and (c) ultrafine grains with the characteristic inferior directionality. Note that the porosity created in the large grains is relatively fine regardless of directionality and that the porosity in the ultrafine-grained sample is comparatively coarse.

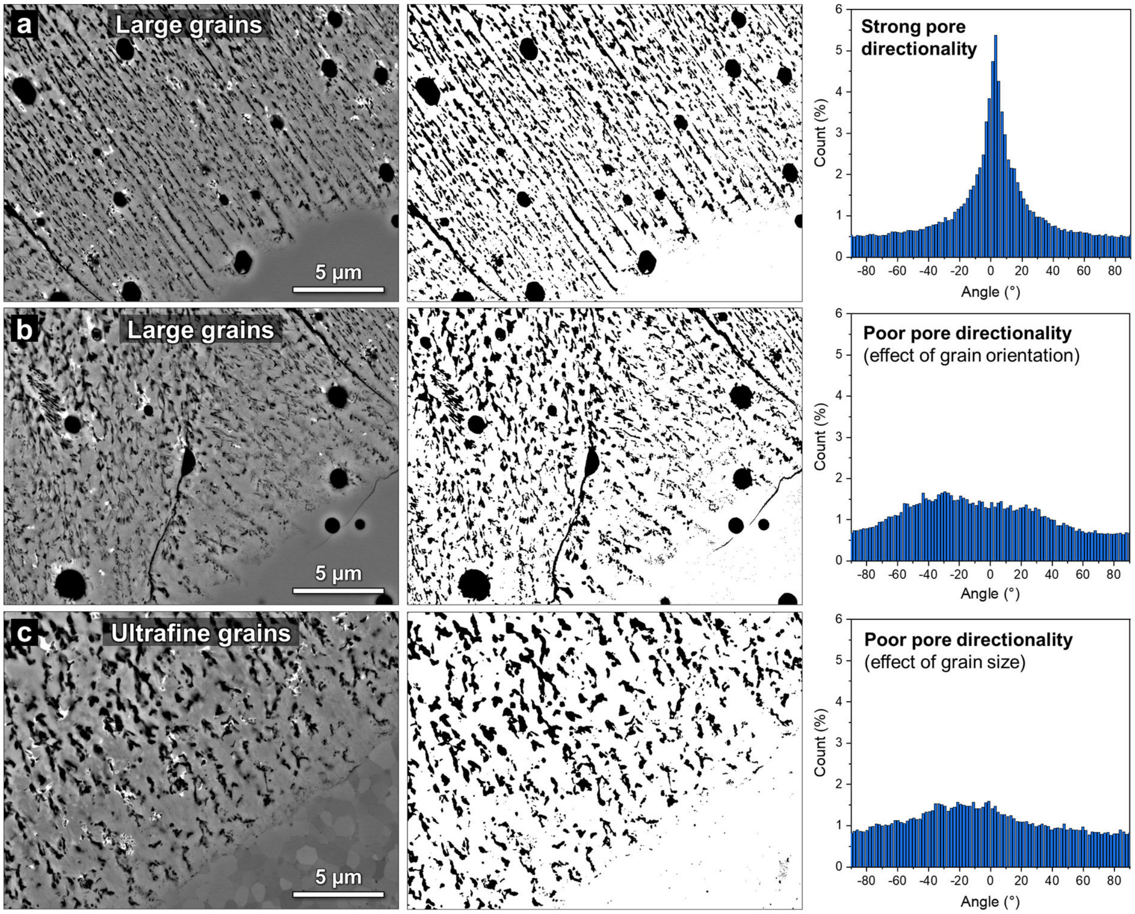

Supplementary Figure 7 | Pore network directionality analysis. BSE-SEM images, image analysis of the porosity and the corresponding angular distribution of pores extended from the hematite/magnetite interface, illustrating the directionality in (a) large-grained region with strong pore directionality, (b) large-grained region with poor pore directionality, and (c) ultrafine-grained sample with poor pore directionality. Note that the deviation angle is calculated relative to the orientation of the interface.

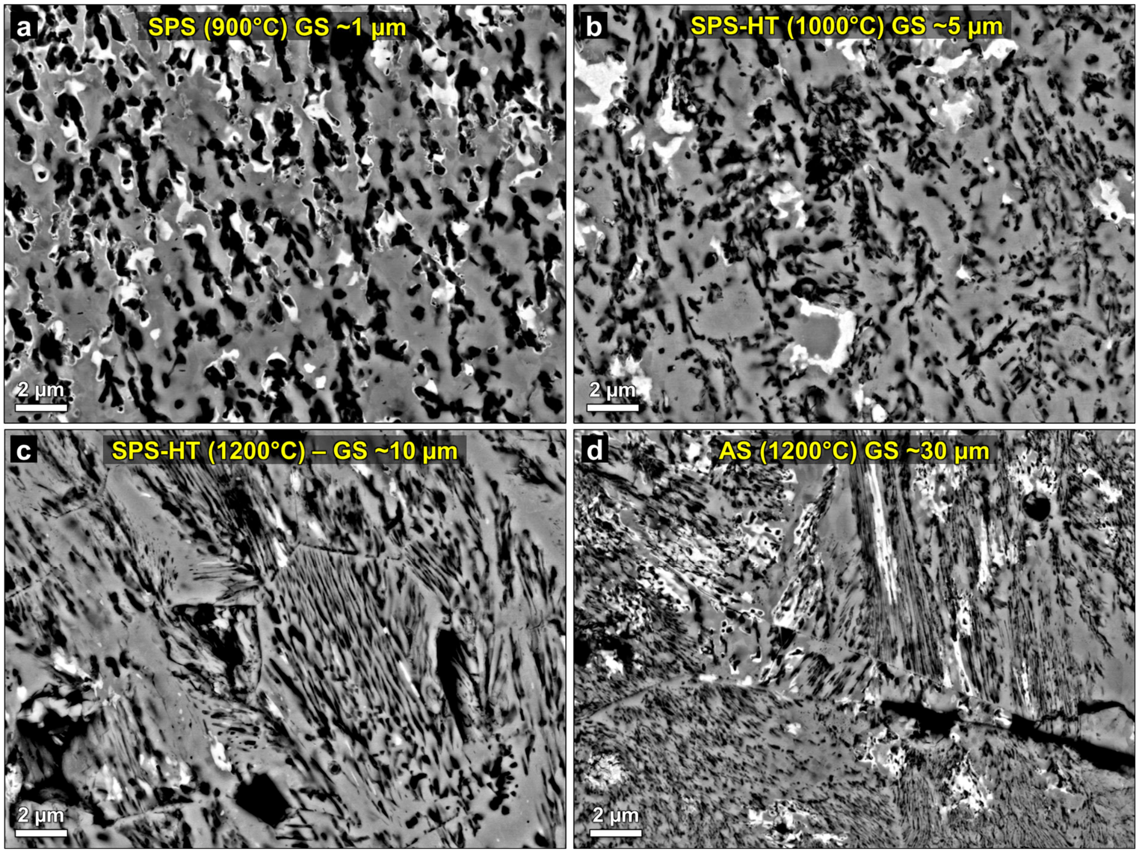

Supplementary Figure 8 | Pore network morphology for different grain sizes. BSE-SEM cross section images taken in partially reduced samples at a distance roughly 30 µm away from the hematite/magnetite interface, showcasing the porosity characteristics depending on grain size. (a) SPS at 900 °C with ultrafine grains size of ~1 µm, (b) SPS + heat treatment at 1000 °C for 6 h with grain size of ~5 µm, (c) SPS + heat treatment 1200 °C for 24 h with grain size of ~10 µm, and (d) air sintered at 1200 °C with a large grain size of ~30 µm.

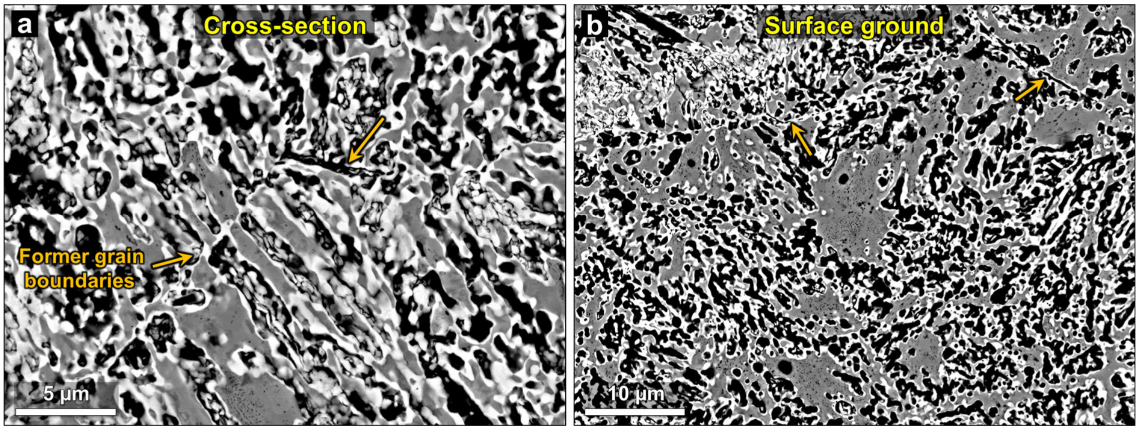

Supplementary Figure 9 | Retained oxides regions. BSE-SEM images of regions in the reduced layer of (a) cross section and (b) ground in surface, showing the typical “trapped” retained oxides that are found in the large-grained sample. Notice that many of these oxides still contain the initial very fine porosity that formed in the magnetite near the hematite. Some of what used to be grain boundaries in the original hematite are marked with arrows.

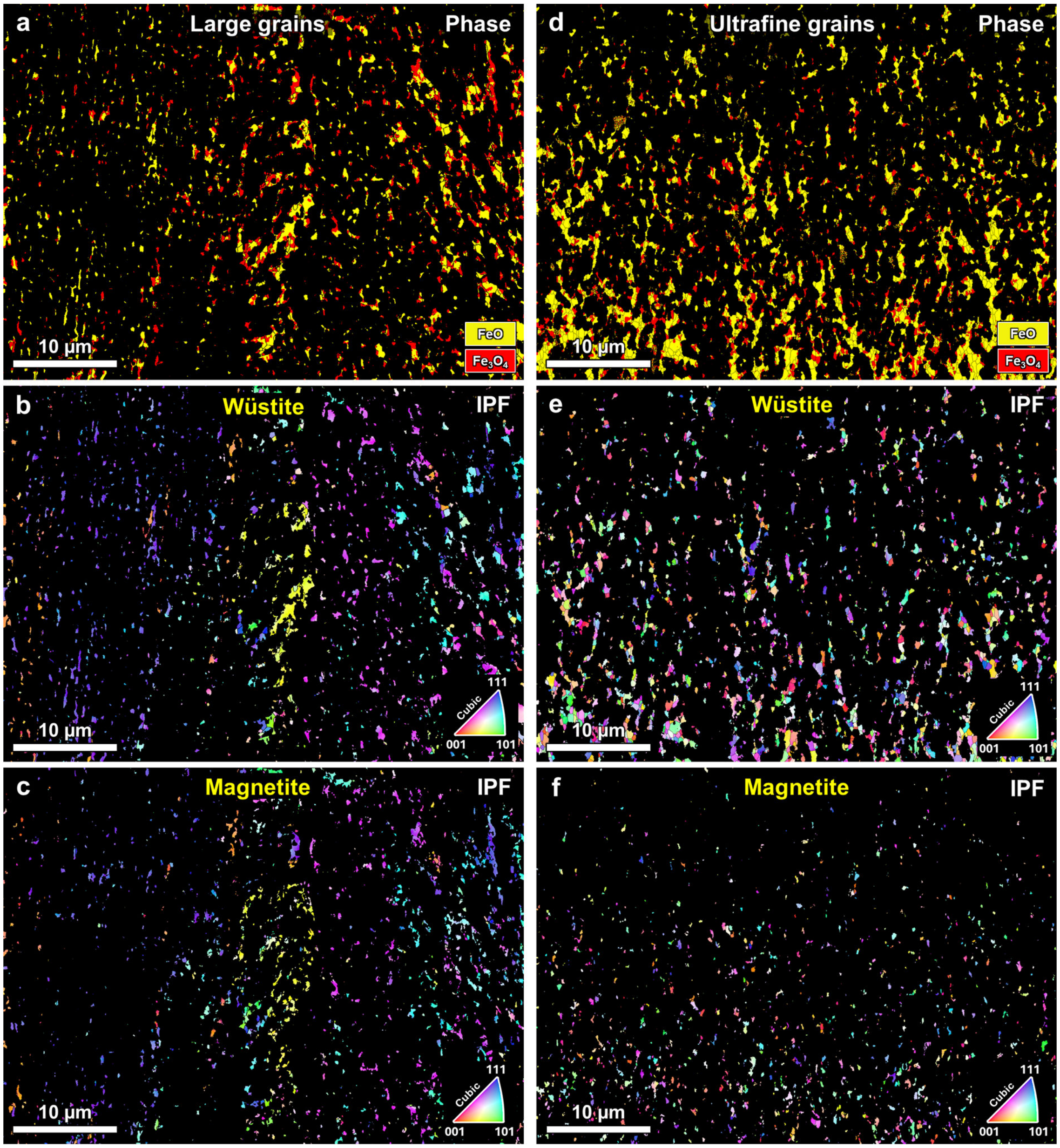

Supplementary Figure 10 | EBSD analysis of the oxide phases in iron-rich regions of the reduced layers. Large-grained sample: (a) phase map, and IPF partitioning of (b) wüstite and (c) magnetite. Ultrafine-grained sample: (d) phase map, and IPF partitioning of (e) wüstite and (f) magnetite. There is evident local texture in the wüstite and magnetite along the reduction direction only in the reduced large hematite grain size sample.

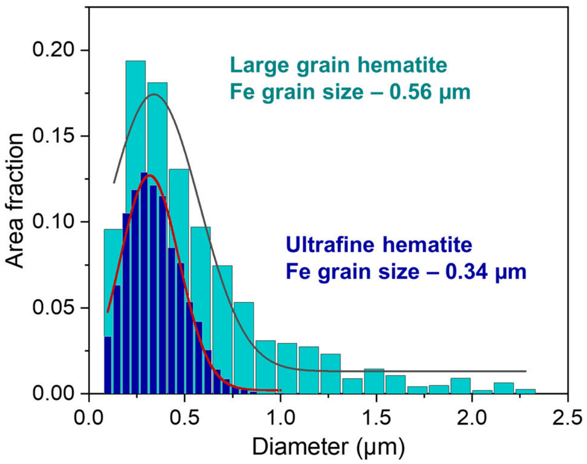

Supplementary Figure 11 | Grain size distribution of Fe grains in the reduced regions of the partially reduced samples that initially had an ultrafine (blue) and large (cyan) hematite grain size.

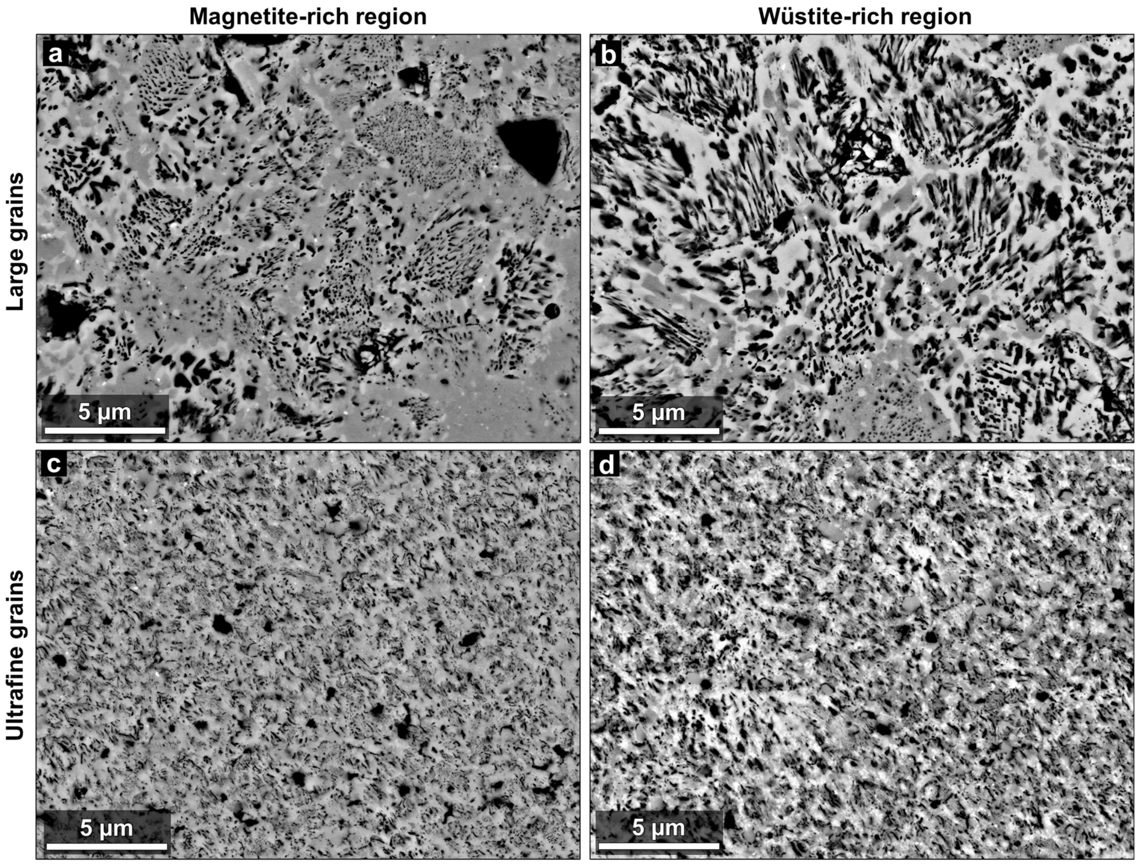

Supplementary Figure 12 | Microstructure of partially reduced samples as seen by grinding into the interface (in the direction of the reduction progression) showing the developed “cell structure” in the oxide-rich regions. Large grains sample at (a) magnetite- and (b) wüstite-rich regions. Ultrafine grains sample at (c) magnetite- and (d) wüstite-rich regions. The darker and lighter grey oxide phases correspond to magnetite and wüstite, respectively.

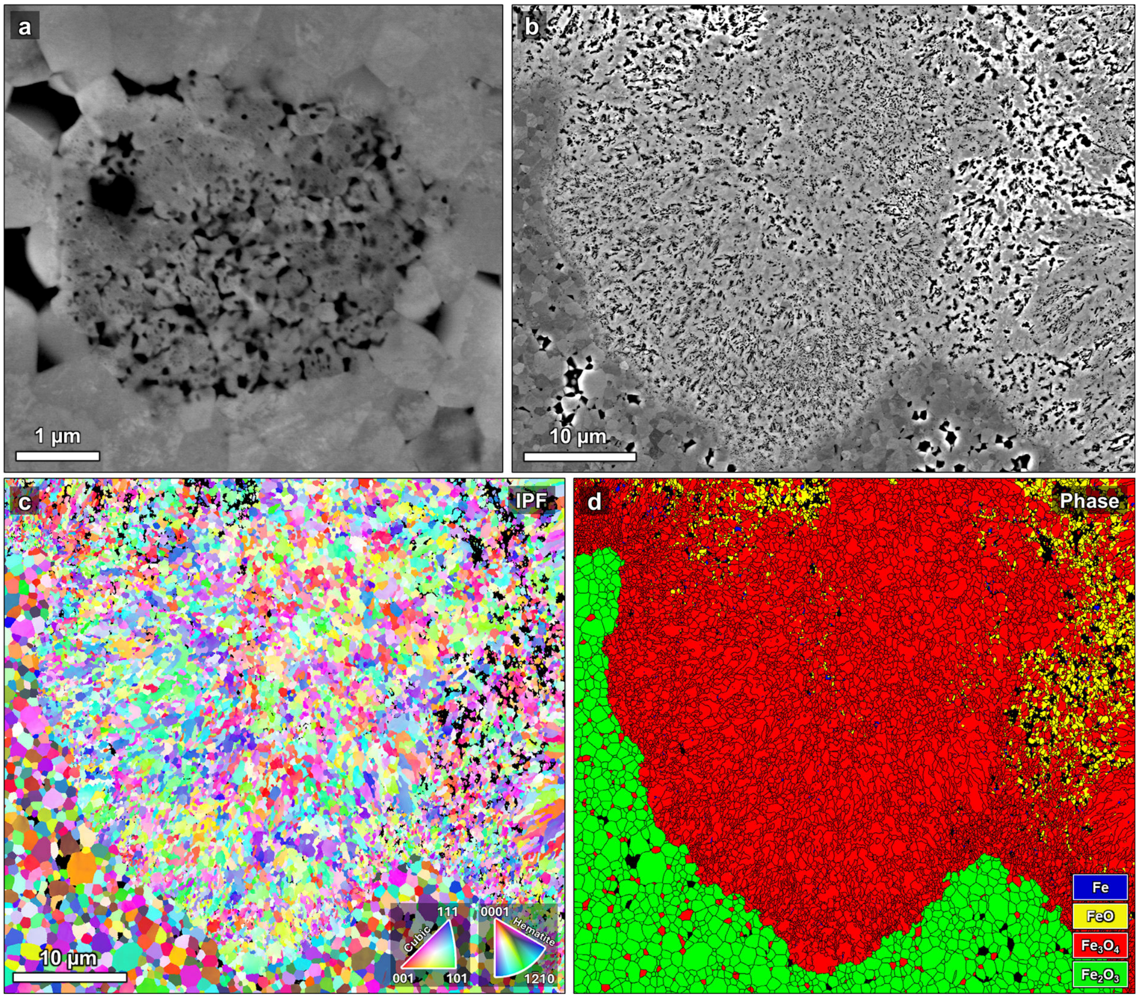

Supplementary Figure 13 | Microstructure of the partially reduced ultrafine-grained hematite ground in from the reduced surface (in the direction of reduction progression). (a) High and (b) low magnification BSE-SEM images showing no discernible "cell structure" in the magnetite at the hematite interface. EBSD analysis (c) IPF and (d) phase map revealing the magnetite structure near the hematite interface.

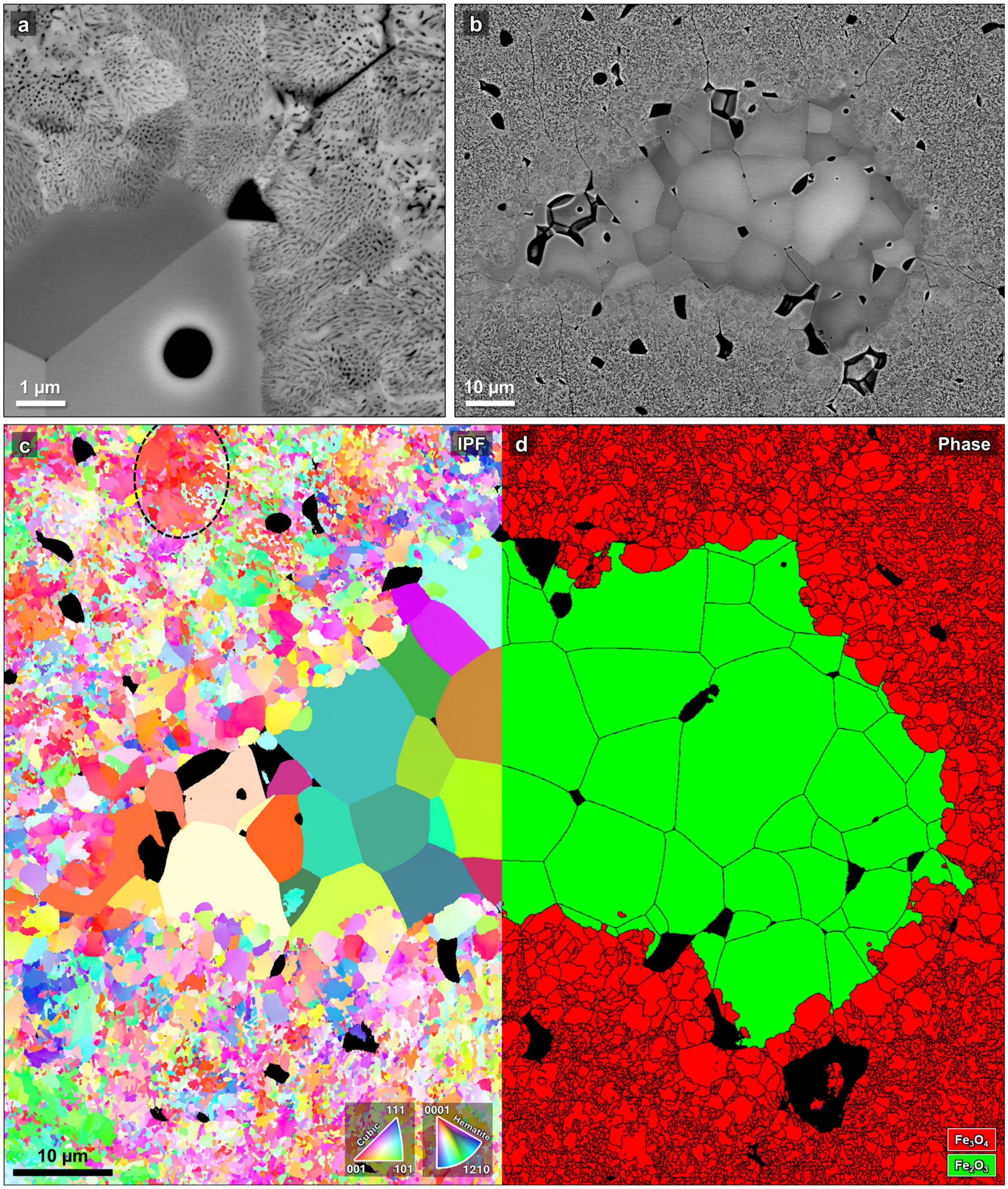

Supplementary Figure 14 | Microstructure of the partially reduced large-grained hematite ground in from the reduced surface (in the direction of reduction progression). (a) High and (b) low magnification BSE-SEM images showing the distinct “cell structure” in the magnetite at the hematite interface. EBSD analysis (c) IPF and (d) phase map revealing the magnetite structure near the hematite interface. Local texturing related to the precursor large hematite grains can be observed, see for example the red region circled with the dashed line in (c). Significant grain refinement is observed following the initially large “cell structure” magnetite grains.
